# Adjuvant-Induced Autoimmune Syndrome: A Bibliometric Analysis

**DOI:** 10.7759/cureus.65184

**Published:** 2024-07-23

**Authors:** Juan E Ospina-Gómez, Maria C Ayala-Gutierrez, Maria C Amaya Muñoz, Catalina Cáceres Ramírez, Edgar F Monsalve-Suárez, Diego L Saaibi-Solano, Paul Anthony Camacho López, Maria G Latorre-Arevalo

**Affiliations:** 1 Research, Development, and Technological Innovation Department, Fundación Oftalmológica de Santander (FOSCAL), Floridablanca, COL; 2 School of Medicine, Universidad Autónoma de Bucaramanga, Bucaramanga, COL; 3 Rheumatology Department, Fundación Oftalmológica de Santander (FOSCAL), Floridablanca, COL

**Keywords:** scopus, bibliometrics, shoenfeld's syndrome, asia syndrome, adjuvant-induced autoimmune syndrome (asia)

## Abstract

The autoimmune/inflammatory syndrome induced by adjuvants (ASIA) encompasses various autoimmune conditions triggered by exposure to substances with adjuvant activity. Despite its potential relevance to public health, global scientific production on ASIA syndrome is significantly limited. This knowledge gap underscores the need for a comprehensive bibliometric assessment to understand global research in this field. Therefore, this article aims to conduct a bibliometric analysis to identify and evaluate research trends related to ASIA syndrome worldwide. A Scopus search identified scientific documents published between 2010 and 2022. A total of 2,133 articles meeting inclusion criteria were selected and analyzed for scientific production, authors, and institutions. Two-hundred fifty six documents were analyzed, mostly journal articles with multiple authors. The year with the highest publications was 2023, marking a notable increase since 2021. Italy and Israel had the most documents and citations, correlating with authors Yehuda Shoenfeld (Israel) and Carlo Perricone (Italy). Standout journals are "The Journal of Immunologic Research" and "Lupus." Relevant affiliations include Tel-Aviv University and the National Autonomous University of Mexico. This article identifies and analyzes scientific trends associated with ASIA syndrome. Despite increased publications, this field remains controversial and lacks full acceptance within the medical and scientific community, as evidenced by limited scientific production compared to other pathologies. These findings may motivate researchers to generate impactful publications, contributing to the global knowledge expansion on this syndrome.

## Introduction and background

The ASIA syndrome, acronym for autoimmune/inflammatory syndrome induced by adjuvants, is a disease entity that was first introduced by Shoenfeld et al. in 2011 as a proposal to categorize autoimmune diseases triggered by environmental factors or adjuvants [1]. This syndrome incorporates five immune-mediated conditions: the post-vaccination phenomena, the macrophagic myofasciitis syndrome (MMF), the Gulf War syndrome (GWS), siliconosis, and the sick building syndrome [1,2]. All these conditions share similar clinical manifestations including myalgia, myositis, arthralgia, neurological manifestations, pyrexia, dry mouth, cognitive alterations, fever, and chronic fatigue syndrome [3]. These shared symptoms are related to a common denominator, which has been subsequently identified in the adjuvant [4]. Adjuvants are compounds that, when introduced into the body, enhance a specific immune response, resulting in higher antibody concentrations [5]. Well-known examples of adjuvants include aluminum hydroxide, squalene, and silica. Over the past decade, it has become evident that human medical implants, including injectables like silicones and polypropylene meshes, can also act as adjuvants [5].

From its initial presentation in 2011 to 2016, more than 4,000 ASIA syndrome cases have been identified, with the majority of severe cases related to vaccines, silicone implants, and mineral oil fillers [6,7]. Moreover, the ASIA syndrome registry (established and managed by the Zabludowicz Center for Autoimmune Diseases at the Chaim Sheba Medical Center) currently contains more than 300 cases of diverse autoimmune conditions, and this number is rising steadily due to the high awareness among physicians worldwide of the existence of this syndrome that has increased significantly in recent years [2,3].

The authors have suggested various major and minor criteria that could assist in identifying ASIA syndrome and facilitate enhanced disease identification and the establishment of standardized definitions. For a condition to be diagnosed as ASIA, two major criteria or one major criterion alongside two minor ones must be fulfilled.

The use of bibliometric analyses serves as a dependable statistical method to measure, compare, analyze, and objectify activities in scientific research. Its primary applications include uncovering emerging trends, collaboration patterns, and exploring the intellectual structure of a specific domain within existing literature [7]. In the context of this emerging pathology, the literature related to ASIA syndrome is extensive, but it has not been mapped in a bibliometric analysis. Hence, the aim of this study is to present a bibliometric analysis of ASIA syndrome research. This analysis not only addresses a conspicuous gap in the scientific literature but also serves as a pivotal reference for future studies on this condition.

## Review

Methods

Search Strategy

The search for articles was carried out in the SCOPUS database, using the following search strategy: "ASIA syndrome" OR "Adjuvant-Induced Autoimmune Syndrome" OR "Auto-inflammatory Syndrome" OR "Autoimmune/inflammatory syndrome induced by adjuvants" OR "Shoenfeld's syndrome" OR “Siliconosis” OR “Mineral oil injection” OR “Post-vaccination phenomena” OR “Macrophagic myofasciitis” OR “Silicone implant incompatibility syndrome”. The literature search was carried out by two authors independently, and then the results were compared. If there were any differences, the authors discussed them with a third author.

Eligibility Criteria

The included documents comprised journal articles, reviews, conferences, books, and book chapters about ASIA. The publication time frame ranged from January 1, 2011, to December 1, 2023, which corresponds to the date of the conducted search. No language restrictions were applied. Book reviews, conference reviews, notes, errata, editorials, letters to the editor, doctoral theses, master's theses, and other non-scientific documents were excluded. Articles that were not available in full text and those involving animal studies were also excluded.

Study Selection and Analysis

The results obtained were exported to a CSV file, including information on bibliographic citations, abstract, keywords, and references. Duplicates and articles excluded by title and abstract were eliminated with the Microsoft Excel® 2016 program (Microsft Corporation, United States). Before selection, a training meeting was held to standardize the definitions, three authors excluded articles by title and abstract independently. For discrepancies, a discussion was held between the two authors. The bibliometric analysis was carried out with Biblioshiny (Bibliometrix) [8], an R package to evaluate scientific production, sources, authors, content, thematic evolution, citation network, joint citation, and collaboration. The publishing trends and tables were analyzed using Microsoft Excel 2016. The tool VOSviewer (v1.6.15, Centre for Science and Technology Studies, Leiden University, The Netherlands) was used for the analysis of keyword co-occurrence [9].

Results

Study Selection

Initially, 2,536 records were identified, 16 duplicate articles and another 245 excluded due to the typology of the article. A total of 2,275 records were evaluated by title and abstract to determine their relevance to the study objective. 

Out of a total of 2019 articles, the majority (1,932) were excluded because they did not reference the ASIA. Although situations that could correspond to the syndrome were described in the title and abstract, the authors did not use the corresponding term of our review. Other reasons for exclusion included studies in animals (58) and articles to which we could not gain access (29).

**Figure 1:**
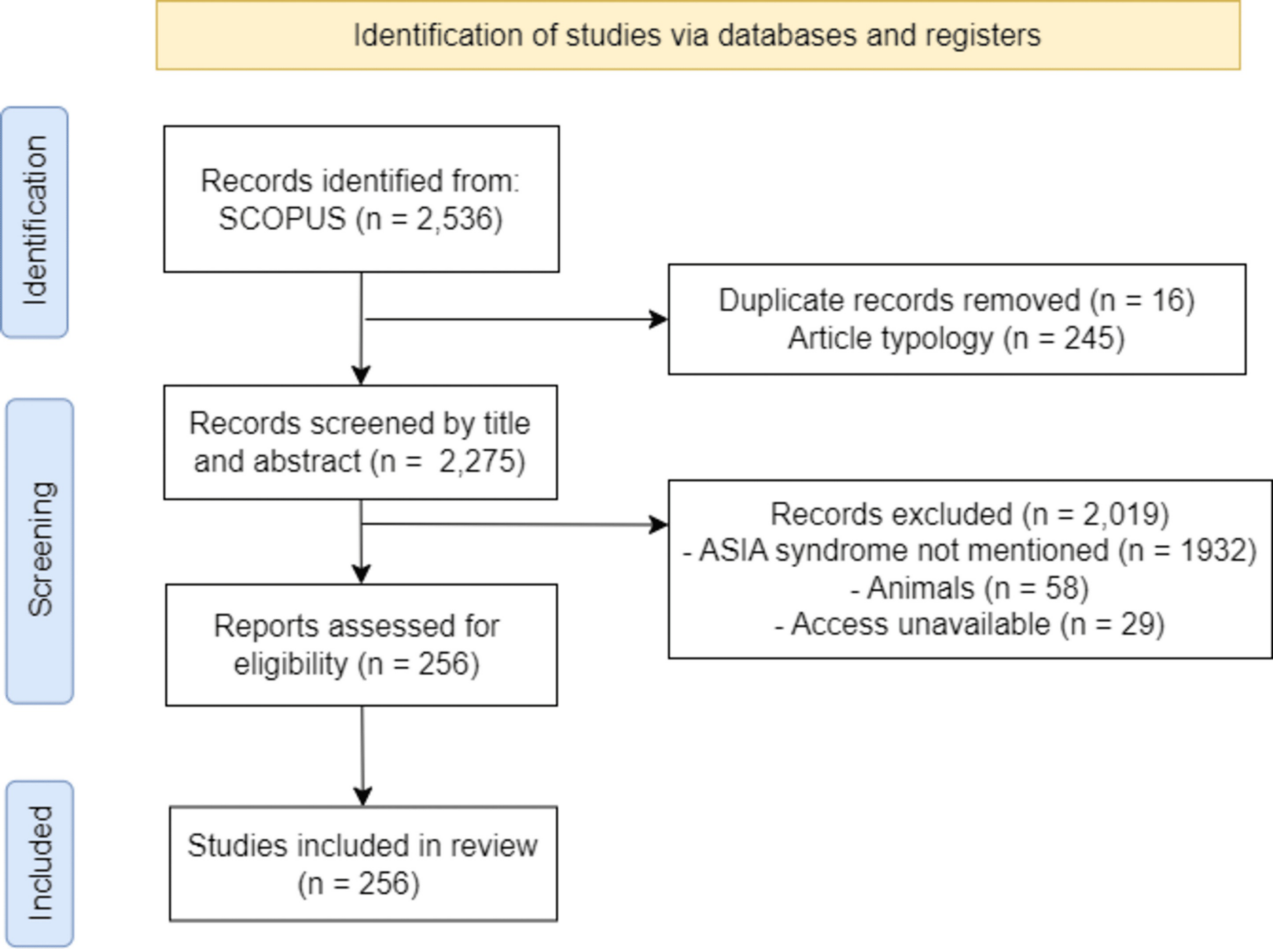
Preferred Reporting Items for Systematic Reviews and Meta-Analyses (PRISMA) flowchart illustrating the item selection process

The final sample for bibliometric analysis was composed of 256 documents that met the inclusion criteria (Figure 1).

Metadata Analysis

Table 1 shows detailed information on the total number of documents included. The majority of these belong to journal articles (62.9%), followed by topic reviews (28.9%). A smaller number of book chapters were also included, along with two conference papers. Of the 928 identified authors, only 15 (1.6%) have single-authored documents. Furthermore, 93.3% of the articles reviewed were published in English, while 3.5% were published in Spanish.

**Table 1:** General information of the articles included

Item	Results
Documents	256
Period	2011-2023
Keywords	601
Authors	
Authors	928
Authors of single-authored docs	15
Types of documents	
Journal articles	161
Book chapter	19
Conference paper	2
Review	74
Languages	
English	239
Spanish	9
Other	8

Temporal Distribution of the Literature

**Figure 2:**
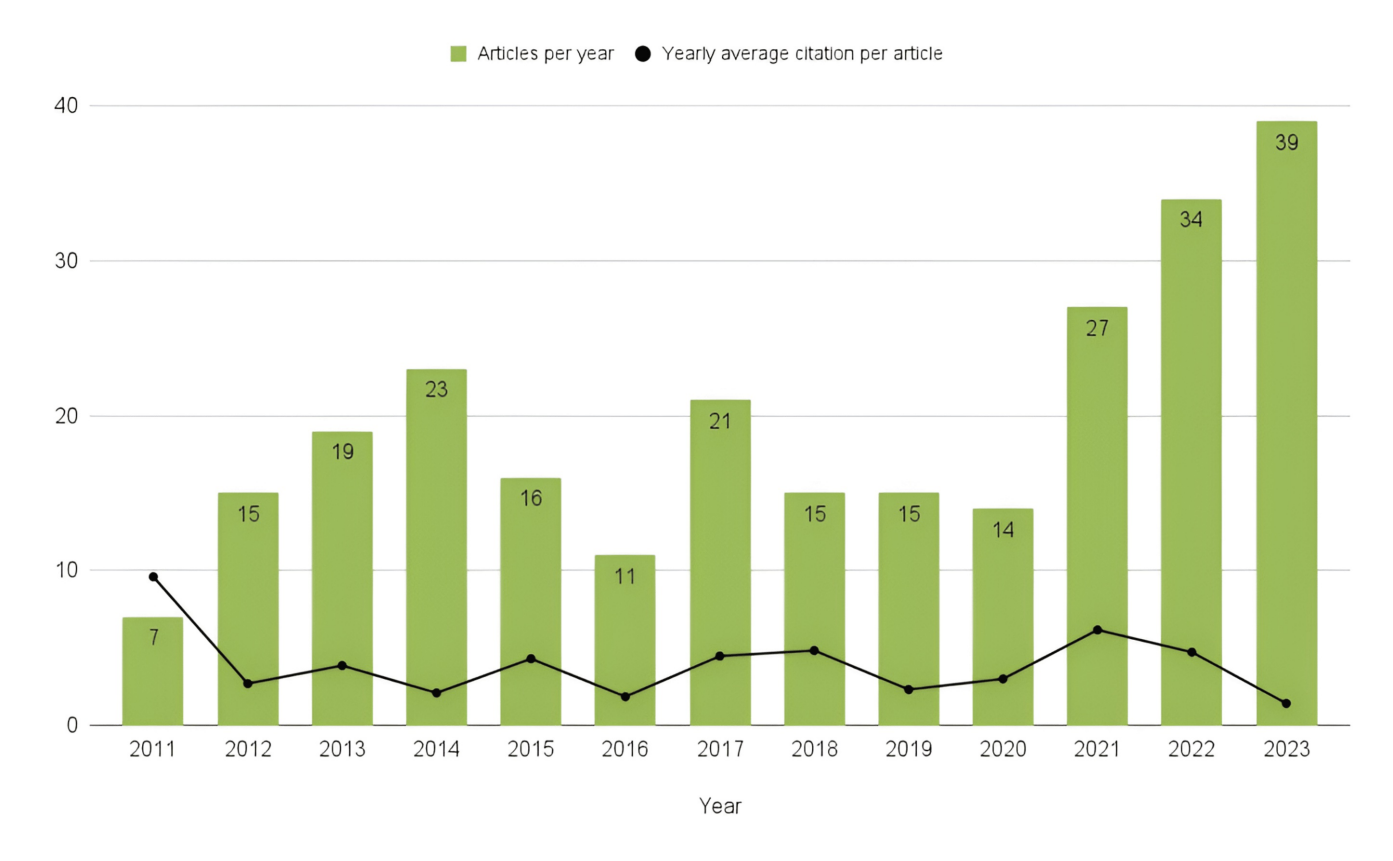
Comparison between scientific production per year and yearly average citation per article

Since 2011, when the term “ASIA syndrome” was introduced, the number of publications has been increasing. After 2020, there is constant growth in research and the use of this term, achieving a productivity of 39 articles published in 2023. By contrast, the yearly average number of times each manuscript has been cited has decreased since 2011. Figure 2 shows that 2011 had the lowest scientific production, but each article was cited approximately nine times. However, in 2012, the scientific production increased to 15 articles, but citations dropped to 2.69 average citations per article. The year 2023 had the highest production, but the articles have not been frequently cited (1.41 average cites per article). 

**Figure 3:**
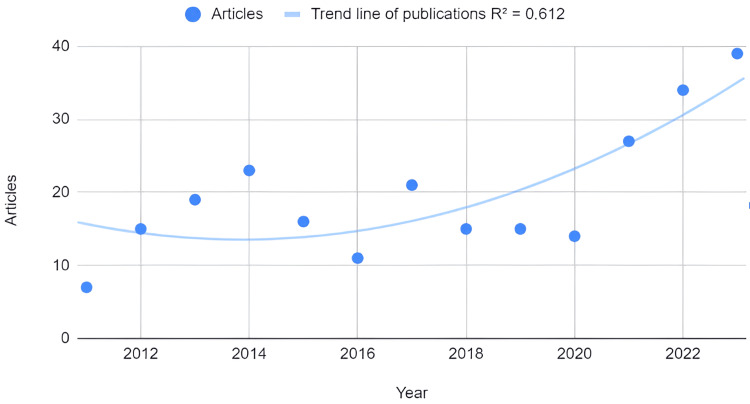
Estimated trend line of publications between years

An estimation of the trend in the number of articles published annually was conducted. The second-order polynomial trendline (1.05E+06 - 1038x + 0.258x^2^) revealed a linear decline in production, as indicated by the negative linear coefficient, coupled with variability in the trend over time. Furthermore, it suggests that approximately 61.2% of the variability in production is not accounted for by this model, indicating the presence of other influencing factors (Figure 3).

Distribution by Country

Table 2 presents the country's scientific production (number of authors appearances by country affiliations), the corresponding author's country, and the total citations per country. In terms of scientific production, a greater contribution is observed from two countries mainly, Israel and Italy. The first stands out as the country with the greatest scientific contributions (188 articles), and the second as the country with the greatest correspondence articles (48 articles) and citations (2,587). Of the 188 authors from Italy who have participated in writing the articles, only 18 are corresponding authors. Mexico stands out as the country with the third place in terms of scientific production (121 articles) and the Netherlands in terms of citations (428). On the other hand, Brazil, despite being among the 10 countries with the highest contributions, has a surprisingly low number of citations compared to other countries. By contrast, the United States has moderate scientific production, but its publications have received a considerable number of citations, indicating a significant influence on research.

**Table 2:** Country's scientific production, author's country, and number of citations

Region	Country's scientific production	Author's country	Number of citations
Italy	188	18	407
Israel	181	48	2587
Mexico	121	15	403
Spain	77	11	242
France	59	11	257
Brazil	54	11	29
Colombia	51	10	42
Netherlands	51	10	428
Turkey	42	7	146
Japan	37	6	24
Poland	23	7	48
USA	18	5	115
India	17	5	11
Portugal	16	2	33
Australia	15	3	71
Canada	15	5	142
New Zealand	6	4	80

**Figure 4:**
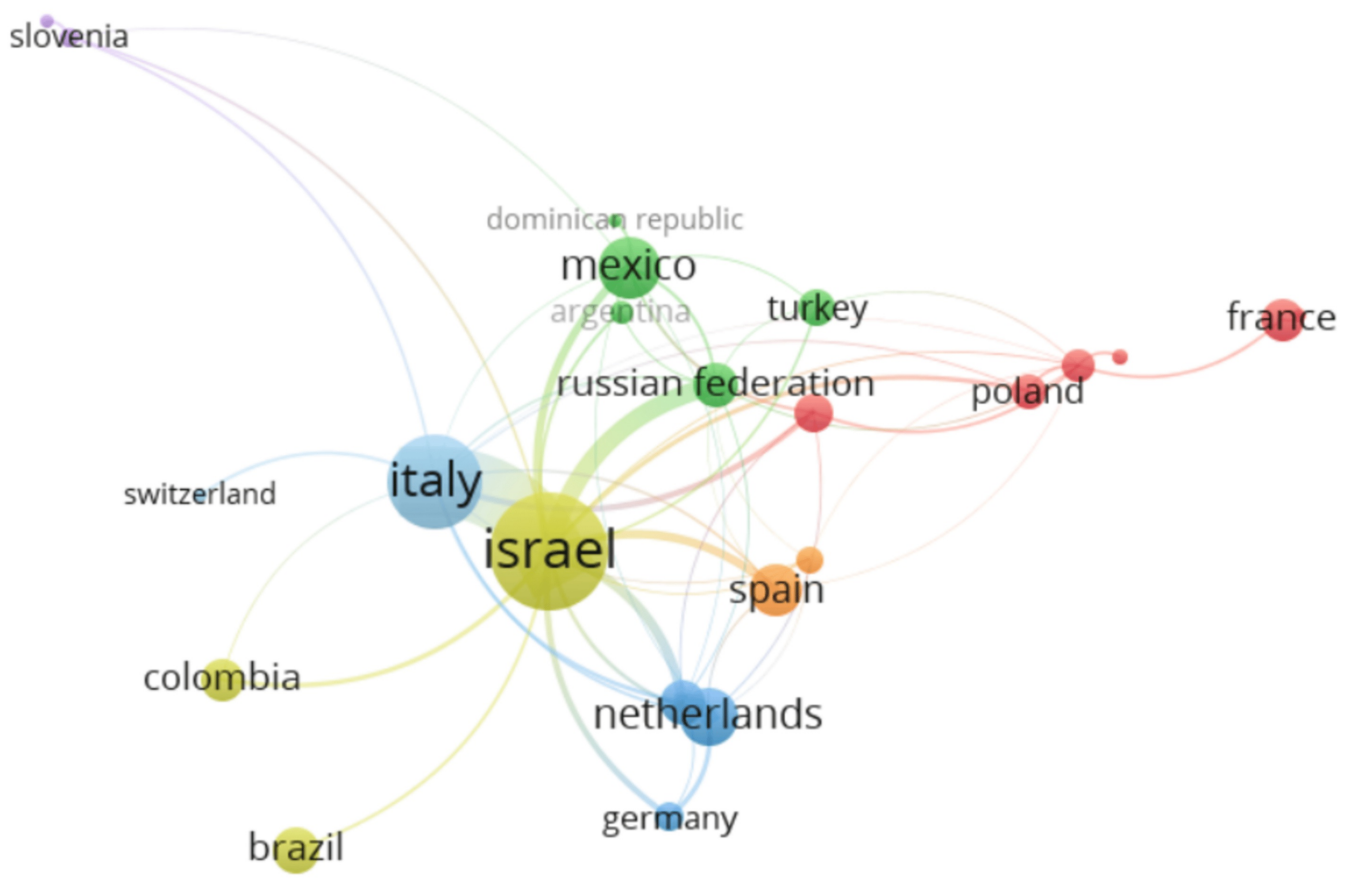
Visual map of the collaboration network between countries Nodes: Each node in the graph represents a country, and its size depends on the number of publications from that country. Connection lines: The lines connecting the nodes represent collaborations between countries, and their width indicates the strength of the collaboration. A wider line suggests a stronger collaboration, representing a higher number of joint publications. Made using VOSviewer

The map of the collaboration network between countries presents 23 of the 47 countries included in the analysis (nodes). The remaining 24 countries do not present collaborations with other countries. The connecting lines between them represent their cooperative relationships. The larger the node, the greater the number of publications, and the wider the line, the stronger the relationships. The countries with the greatest international collaboration are Israel (15 collaborations), Italy (13), Mexico (10), and the United Kingdom (10). Israel and Italy had the strongest cooperation of the group (link strength: 22.45). Other strong relationships were between Israel with Russia (link strength: 6.03), Canada (link strength: 3.17), and Spain (link strength: 2.78) (Figure 4).

Author Distribution

Among the most relevant authors in the field of ASIA syndrome, the work of Yehuda Shoenfeld stands out. Shoenfeld is the founder and director of the Zabludowicz Center for Autoimmune Diseases, Sheba Medical Center, in Israel. He is recognized for having introduced the term "ASIA syndrome" for the first time and has 59 documents in this revision, being the author with the highest impact factor (h-index = 26).

On the other hand, Italian rheumatologist Carlo Perricone is positioned in second place in the number of published documents and impact factor, with 15 articles identified in this review and an h-index of 11.

In the third place, in the number of published documents and second in the impact index (with Perricone), is Howard Amital, from the Department of Internal Medicine of the Autoimmune Disease Research Center at Sheba Medical Center, who has 14 articles in this analysis, and an h-index of 11.

Other relevant authors in terms of scientific production are Jara LJ, Agmon-Levin N, Cohen Tervaert JW, Watad A, Vera-Lastra O, Medina G, and Bragazzi NL. It is notable that of the 10 authors exhibited, four are from Israel, two are from Italy, three are from Mexico, and one is from the Netherlands (Table 3).

**Table 3:** Most relevant authors according to impact, publications, affiliation, and country

Author	Articles	H-index	Affiliation	Country
Shoenfeld Y	59	26	Zabludowicz Center for Autoimmune Diseases, Sheba Medical Center	Israel
Perricone C	15	11	Rheumatology Unit, Department of Medicine and Surgery, University of Perugia	Italy
Amital H	14	11	Zabludowicz Center for Autoimmune Diseases, Sheba Medical Center, and Tel Aviv University	Israel
Jara LJ	13	9	Universidad Nacional Autónoma de Mexico	Mexico
Agmon-Levin N	12	9	Center for Autoimmune Diseases and Department of Medicine B, Sheba Medical Center	Israel
Cohen Tervaert JW	12	9	Maastricht University	Netherland
Watad A	11	10	Zabludowicz Center for Autoimmune Diseases, Sheba Medical Center, and Tel Aviv University	Israel
Vera-Lastra O	11	7	Universidad Nacional Autónoma de Mexico	Mexico
Medina G	9	7	Universidad Nacional Autónoma de Mexico	Mexico
Bragazzi NL	7	6	University of Genoa	Italy

Most Relevant Sources

**Figure 5:**
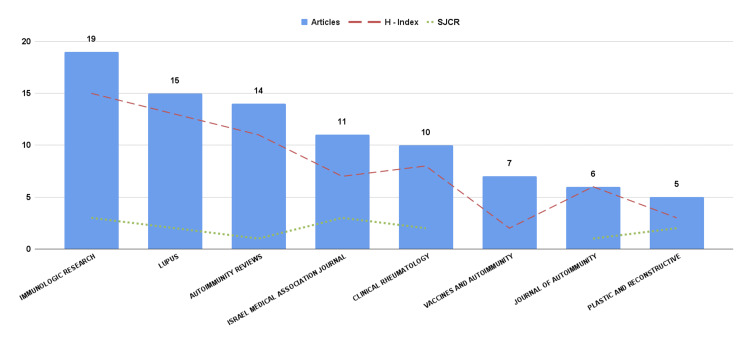
Number of articles published and impact index per journal SJRC: Scimago Journal & Country Rank

Figure 5 presents the eight journals that published five or more articles related to ASIA syndrome and that are found in zone one of Bradford, highlighting the concentration of scientific production in these specialized sources. Six of these journals are related to immunology, one to medicine in general and another to plastic surgery. The journal *Immunologic Research* (Q3) stands out as the source with the highest number of articles (19) and the highest impact index (h-index) (15). Followed by Lupus, with 15 articles, a local h-index that is also high (13) and is located in quartile two (Scimago Journal & Country Rank (SJCR)). Journals such as *Autoimmunity Reviews*, *Israel Medical Association Journal,* and *Clinical Rheumatology* have 14, 11, and 10 publications, respectively.

Affiliation Analysis

Among the institutions with the highest number of scientific publications, Tel Aviv University (Israel) stands out, leading with a total of 105 articles. The National Autonomous University of Mexico is in second place with 37 publications. The Zabludowicz Center for Autoimmune Diseases in Israel has contributed a total of 28 scientific articles on this topic. Sapienza University of Rome has a total of 22 publications, followed by St. Petersburg State University (21), Maastricht University (17), University of Brescia (15), and Istanbul Medipol University (14) (Table 4).

**Table 4:** Number of articles published by affiliation

Affiliations	Country	Articles
Tel Aviv University	Israel	105
National Autonomous University of Mexico	Mexico	37
Zabludowicz Center for Autoimmune Diseases	Israel	28
Sapienza University of Rome	Italy	22
St. Petersburg State University	Russia	21
Maastricht University	Netherlands	17
University of Brescia	Italy	15
Istanbul Medipol University	Türkiye	14

Most Frequent Words

**Figure 6:**
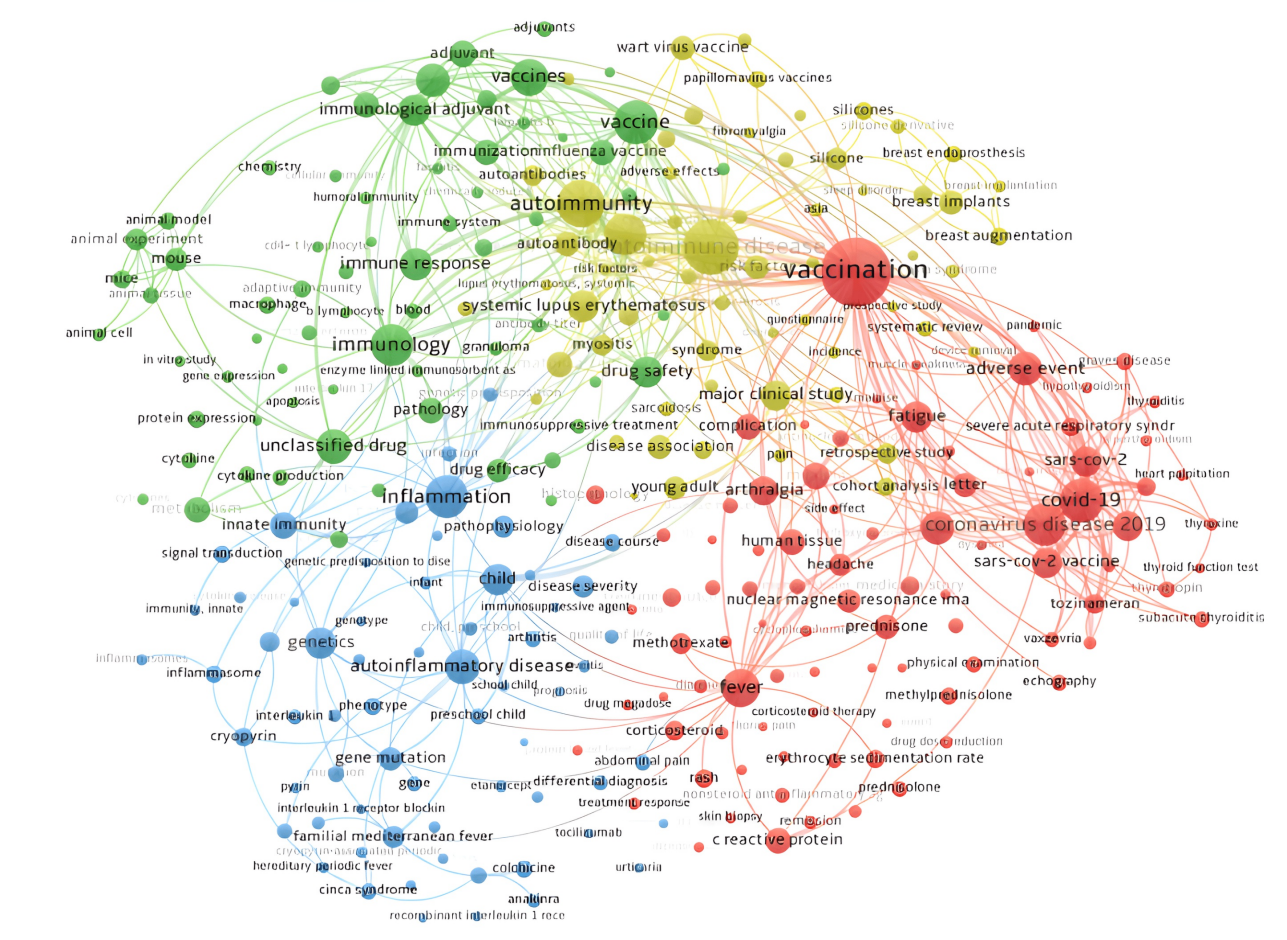
Most frequent keywords and associations This figure presents the most frequent words in the included articles, identifying the main themes of the review. The size of each node reflects the frequency of the corresponding keyword, and the distance between nodes indicates the strength of their relationship. Red group: focuses on terms related to Covid-19 and its symptoms, as well as the impact of vaccines as potential adjuvants causing ASIA syndrome. Yellow group: shows a strong linkage between autoimmunity issues and adjuvants. Green group: focuses on the immunological reactions underlying the pathophysiology of ASIA syndrome. Blue group: related to inflammation and disease pathophysiology. Made using VOSviewer

Figure 6 presents the most frequent words of the included articles, which allows the main themes of the review to be identified. The size of the nodes is a function of the frequency of each of the keywords, and the distance between them indicates the strength of the relationship. The most frequent keywords were grouped into four groups according to their relationships.

The group identified in red mainly highlights key terms such as "vaccination," "COVID," "SARS-CoV-2," and "fever." This pattern reflects the relevance of COVID-19 disease and its connection with symptoms such as fever, which is also associated with ASIA syndrome. In addition, the presence of vaccines stands out, which have been identified as adjuvants causing the syndrome.

In the yellow group, a strong relationship is observed between autoimmunity and adjuvants such as “silicones”, “breast implants,” and “HPV vaccines." This group shows a close relationship with the green group, which focuses on the pathophysiology of the immunological reaction of ASIA syndrome, and relates words such as “immunology," “vaccine," and “cytokine.” This group is linked to the blue group, whose key terms are also related to inflammation and the pathophysiology of the disease, highlighting words such as “innate immunity," “inflammatory diseases," and “genetic mutations."

Discussion

This bibliometric analysis represents a significant contribution to the field of ASIA. To the best of our knowledge, this study is the first to provide a bibliometric analysis of published ASIA syndrome research worldwide. We identified countries, institutions, and journals in ASIA syndrome with the highest number of publications and citations, as well as the most-cited research articles, keywords, and patterns of research collaboration. Furthermore, it evaluates a period of 12 years from the first description of the syndrome to the present.

Within different types of research articles, bibliometric analysis evaluates the current state and trends of the literature in a specific research domain [10]. This allows to highlight areas of importance and potential gaps in the literature, helping to provide ideas and directions for future research.

The results show that between 2011 and 2023, approximately 2,536 documents related to the topic were published, with a sustained growth trend. The heightened awareness of ASIA syndrome stems from increased understanding of its concepts and extensive research on the link between different adjuvants and autoimmune reactions over recent years [11]. Since its emergence as a disease entity, more than 4,000 documented cases of ASIA syndrome have been reported [6].

Using a second-order polynomial model, 61% of the variability remains unexplained by temporal trends alone, indicating diverse factors influencing scientific production in this field. Socioeconomic dynamics, technological advancements, changes in disease awareness, international collaborations, and evolving definitions contribute to increased scientific output and variability in trends.

The primary form of scientific production for ASIA syndrome is through journal articles. Case reports comprise the largest portion of scientific information, offering insights into pathologies with limited existing literature and serving as a significant source of information on ASIA syndrome.

Of the total articles found in the search, it was found that 53 correspond to case reports. The most cited case report corresponds to “Silicone Implant Incompatibility Syndrome (SIIS): A Frequent Cause of ASIA (Shoenfeld's Syndrome)” by authors J W Cohen Tervaert and R M Kappel [12]. This case report represents one of the earliest analyses of the immune system's response to silicone-filled breast implants, proposing a connection between symptoms and implant aging or rupture.

Italy and Israel stand out as leading contributors to scientific production on ASIA syndrome, with 188 and 181 publications, respectively. This can be attributed partly to Yehuda Shoenfeld, an Israeli researcher credited with first describing ASIA syndrome [13]. Collaborations with Italian authors, such as rheumatologist Carlo Perricone [14], also contribute significantly to this output. The comparable scientific production between Italy and Israel likely stems from joint efforts and collaborative publications, exemplified by works like "Autoimmune/Inflammatory Syndrome Induced by Adjuvants and Sjögren’s Syndrome" [15].

It is essential to elucidate that, despite Italy exhibiting a higher volume of publications, its citation count is 407, positioning it in third place. By contrast, Israel has a total of 2,587 citations. This discrepancy may be attributed to the origin of the term ASIA, introduced by Shoenfeld et al. in 2011. Notably, Shoenfeld is the founder and director of the Zabludowicz Center for Autoimmune Diseases and serves as the President of the Sheba Medical Center, affiliated with the Sackler School of Medicine at Tel Aviv University in Israel.

The importance of publishing in high-quality journals cannot be overstated, as it directly impacts the visibility and citation metrics of scientific articles, as measured by the H-index. Notably, journals like *Immunologic Research* [16], *Lupus* [17], and *Autoimmunity Reviews* [18] have the highest publication rates in this field. These journals are based in the United States, the United Kingdom, and the Netherlands, respectively, underscoring their international reach.

In this review, Yehuda Shoenfeld has the most significant scientific output, credited with introducing the term "ASIA syndrome" and having authored over 1,750 articles. The analysis identified 59 documents authored by him, being the author with the highest impact factor (H-index = 26). Shoenfeld also serves as the editor-in-chief of the scientific journal *Autoimmunity Reviews*. Italian rheumatologist Carlo Perricone ranks second in number of published documents and impact factor, with 15 articles identified in this review and an H-index of 11.

The correlation between impact factor (or H-index) and high scientific production is evident. Author Shoenfeld, with the highest production and impact factor, suggests that most of their articles are widely read and cited, indicating good quality and significant impact. 

Regarding the keywords, it is crucial to know them as they provide a guide and orientation for directing future research. The keywords in this article are related to one of the main causes of ASIA syndrome, which is vaccination. In addition, other terms such as "autoimmune diseases" and "immunology" are highlighted, correlating with the studied disease, which, by its acronym, corresponds to an autoimmune syndrome. Finally, "inflammation" is included, which is also related to the studied disease.

Vaccination and ASIA Syndrome

In this bibliometric analysis, the most recurrently associated keyword with ASIA syndrome is "vaccination," reflecting the relationship between vaccination and ASIA syndrome, including SARS-CoV-2 vaccine. The autoimmune-related effects of vaccines were well-documented before the formal recognition of ASIA syndrome [19]. ASIA syndrome provided a unified term for diverse vaccine side effects, advocating for the development of immune stimulators as alternatives to adjuvants [19].

Adjuvants encompass a range of materials, from aluminum-based substances commonly used in vaccines to silicon and heavy metals like mercury and iodine gadital, which have been associated with immune responses [6]. The hepatitis B vaccine is notably associated with the highest frequency of autoimmune manifestations among existing vaccines [20]. Furthermore, several other vaccines have been linked to autoimmune disorders, including reported cases of immune thrombocytopenic purpura [21] and diabetes mellitus [22] following measles, mumps, and rubella (MMR) vaccination.

In this study, we encountered several limitations. Although we used a large database, our results may not fully represent all ASIA syndrome research. We did not explore semi-published literature databases, which might have provided more records, but their reliability was uncertain. In addition, many articles were excluded based on study design criteria, aiming to prioritize scholarly journals with reliable information and rigorous methodologies.

This research holds significant international implications as the first global bibliometric analysis of scientific production related to ASIA syndrome. It offers valuable insights for decision-makers to bolster public health policies. Despite identifying a substantial number of articles, ASIA syndrome remains relatively understudied by researchers. Therefore, the goal is to encourage more authors to contribute to this field and increase scholarly article output. 

Researching the ASIA syndrome reveals a connection between autoimmune and inflammatory conditions and certain exogenous factors, such as adjuvants, intrinsic to the expertise of rheumatologists. Understanding research trends in this domain contributes to identifying new triggering factors emerging from research, enabling the development of prevention and early intervention strategies. In addition, the frequency and clinical characteristics of rheumatological diseases associated with the ASIA syndrome vary among different regions worldwide, along with research and scientific advancements. Thus, comprehending these differences allows recognition of leading countries and institutions to support future research. Furthermore, establishing collaborations with experts in the field fosters a broader understanding of the ASIA syndrome and its implications for rheumatology practice globally.

## Conclusions

This article facilitates the identification and analysis of scientific trends in ASIA syndrome. Despite a growing number of publications, this field remains controversial and is not fully embraced by the medical and scientific community, as indicated by its limited scientific output compared to other pathologies. Furthermore, this study can serve as a reference for detecting emerging research areas, influential authors, and significant affiliations within the field.
